# Review on Emerging Therapeutic Strategies for Managing Cardiovascular Disease

**DOI:** 10.2174/011573403X299265240405080030

**Published:** 2024-04-16

**Authors:** Minal Narkhede, Avinash Pardeshi, Rahul Bhagat, Gajanan Dharme

**Affiliations:** 1 SMBT College of Pharmacy, Nandi Hills Dhamangaon Taluka Igatpuri, Nashik 422403, India

**Keywords:** Cardiovascular disease, hypertension, blood vessels, artificial intelligence, traditional medicines, heart attack

## Abstract

Cardiovascular disease (CVD) remains a foremost global health concern, necessitating ongoing exploration of innovative therapeutic strategies. This review surveys the latest developments in cardiovascular therapeutics, offering a comprehensive overview of emerging approaches poised to transform disease management. The examination begins by elucidating the current epidemiological landscape of CVD and the economic challenges it poses to healthcare systems. It proceeds to scrutinize the limitations of traditional therapies, emphasizing the need for progressive interventions.

The core focus is on novel pharmacological interventions, including advancements in drug development, targeted therapies, and repurposing existing medications. The burgeoning field of gene therapy and its potential in addressing genetic predispositions to cardiovascular disorders are explored, alongside the integration of artificial intelligence and machine learning in risk assessment and treatment optimization.

Non-pharmacological interventions take center stage, with an exploration of digital health technologies, wearable devices, and telemedicine as transformative tools in CVD management. Regenerative medicine and stem cell therapies, offering promises of tissue repair and functional recovery, are investigated for their potential impact on cardiac health.

This review also delves into the interplay of lifestyle modifications, diet, exercise, and behavioral changes, emphasizing their pivotal role in cardiovascular health and disease prevention. As precision medicine gains prominence, this synthesis of emerging therapeutic modalities aims to guide clinicians and researchers in navigating the dynamic landscape of cardiovascular disease management, fostering a collective effort to alleviate the global burden of CVD and promote a healthier future.

## 1 INTRODUCTION

### 1.1 Background and Significance of Cardiovascular Disease

CardioVascular disease (CVD) remains one of the prominent causes of infirmity and death globally, imposing a major public health problem. CardioVascular disease comprises a range of circumstances affecting the blood vessels and heart, as well as heart, coronary artery disease, stroke, failure, and PVD. These conditions are frequently related to risk aspects such as hypertension, smoking, high cholesterol, obesity, and diabetes. The incidence of CardioVascular disease continues to increase, with a considerable influence on the worth of life for affected entities and substantial healthcare expenses for society [1, 2]. Descriptions related to CVDs are given in Table **1**.

The significance of CVD lies not only in its high prevalence but also in its extensive consequences. CardioVascular events, such as strokes and heart attacks, can lead to decreased, life expectancy disability, and a moderated value of life for patients and their families. This burden extends to the healthcare system, straining resources and posing challenges to healthcare professionals in terms of diagnosis, prevention, and treatment [3, 4].

### 1.2 Brief Overview of the Current Treatment Approaches

Traditional treatment approaches for CardioVascular disease primarily focus on risk factor organization and indication relief. Medicines such as beta-blockers, statins, and ACE inhibitors, are prescribed to regulate blood pressure, lower cholesterol levels, and manage symptoms. Lifestyle modifications including exercise, dietary changes, and smoking termination, play a crucial role in preventing and managing CVD. In more severe cases, surgical mediations like bypass surgery angioplasty are used to re-establish blood flow to the heart or alleviate blockages in the arteries [6, 7].

While these approaches have been effective in reducing CardioVascular events and improving patient outcomes, there is a need for more targeted strategies and innovative, especially for individuals who do not respond optimally to existing treatments. Moreover, the rising prevalence of CVD and the related costs on healthcare systems emphasize the urgency of exploring new therapeutic options that can further enhance the management of CVD [8].

### 1.3 Purpose of the Review: To Explore Emerging Therapeutic Strategies

The prime objective of this review is to delve into the latest developments and emerging therapeutic strategies for managing CardioVascular disease. As we confront an aging population and an increase in CVD cases, it is essential to investigate new and innovative approaches that hold promise for increasing patient outcomes and decreasing the overall burden of the disease.

This review will provide a comprehensive analysis of cutting-edge therapeutic approaches, including genetic and personalized medicine, immunotherapies, nanotechnology and drug delivery, stem cell therapy, telemedicine, artificial intelligence, lifestyle and behavioural interventions, gene editing, and more. By exploring these emerging strategies, we aim to inform healthcare professionals, researchers, and policymakers about the potential breakthroughs that could shape the future of CardioVascular disease management.

In the subsequent sections of the review, we will examine each of these emerging therapeutic strategies in greater detail, summarizing recent developments, clinical trials, and their potential impact on the field of CardioVascular medicine.

## 2 RISK FACTORS AND EPIDEMIOLOGY

### 2.1 **Identification of Cardiovascular Disease Risk** Factors

CardioVascular disease (CVD) is a multifactorial situation, and the identification of risk factors is serious for controlling and prevention. Common risk factors for CardioVascular disease include:

1. **Hypertension:** For CardioVascular Disease leading risk factor is High blood pressure. It sets stress on the and blood vessels heart, accumulates the risk of heart attacks, strokes, and further CardioVascular events [9].
2. **High Cholesterol:** Raised amounts of low-density lipoprotein cholesterol, often stated as “bad” cholesterol, contribute to the growth of atherosclerosis, a situation where arteries become hardened and narrowed [10].
3. **Smoking:** Tobacco usage is a foremost reason for preventable deaths and is sturdily related to CVD. Smoking harms the blood vessels and speeds up atherosclerosis [11].
4. **Obesity:** Excess body weight, mostly abdominal obesity, is related to CVD. It can lead to high blood pressure, insulin resistance, and adverse lipid profiles [12].
5. **Diabetes:** Equally diabetes mellitus and insipidus are risk factors for CardioVascular Disease. High blood glucose levels can harm blood vessels and nerves over the period, and increase the possibility of heart disease [13].
6. **Physical Inactivity:** The absence of regular physical movement is a risk factor for CardioVascular Disease. Physical activity helps uphold a healthy weight, control blood pressure, and recover general CardioVascular health [14].
7. **Unhealthy Diet:** Diets with saturated and trans fats, salt, and added sugars can lead to hypertension, obesity, and high cholesterol, aggregate the possibility of CardioVascular Disease [15].
8. **Family History:** A family background of CVD, exclusively if it includes a first-degree family member (parent or sibling), can rise an individual's risk [16].
9. **Stress:** Prolonged stress and certain mental health disorders are connected with CVD. Stress may lead to unhealthy handling behaviours identical to overeating or smoking [17].
10. **Excessive Alcohol Consumption:** Alcohol Consumption in large volumes can lead to heart muscle damage, high blood pressure, and irregular heart rhythms, all of which can lead to CVD [18].

### 2.2 Prevalence and Global Burden of Cardiovascular Disease

The global burden of CardioVascular disease is substantial, and its prevalence continues to rise. Key epidemiological factors include:

1. **Global Prevalence:** CardioVascular disease is a global health challenge, affecting individuals of all ages and backgrounds. It is accountable for a major proportion of all deaths worldwide [19].
2. **Mortality Rates:** CardioVascular Disease is the most important cause of mortality worldwide. It is responsible for a substantial portion of deaths in both high-income and low- to middle-revenue countries [20].
3. **Economic Impact:** The economic burden of CVD is huge, surrounding not only healthcare costs but also lost productivity and reduced quality of life. It places a strain on healthcare systems and societies [21].
4. **Age and Gender Disparities:** While CVD risk increases with age, it is not limited to the elderly. Younger individuals can also be affected. Additionally, there are gender disparities in the prevalence and presentation of CVD, with some conditions being more common in women, while others are more prevalent in men [22, 23].
5. **Geographic Variations:** The burden of CVD varies by region, with certain areas having higher rates of specific risk factors. Urbanization and changes in lifestyle contribute to these variations [24].
6. **Preventability:** Many cases of CVD are preventable through lifestyle modifications and risk factor management. Successful prevention and initial intervention approaches are essential to decreasing the burden of the disease [25].

Understanding the risk factors and global epidemiology of CVD is crucial for implementing public health initiatives and targeted interventions to reduce the incidence and impact of CardioVascular disease on individuals and society.

## 3 TRADITIONAL TREATMENT APPROACHES

### 3.1 Medications (*e.g*., Statins, Beta-Blockers, ACE Inhibitors)

Traditional pharmacological interventions have played a central role in managing CardioVascular disease. Several classes of medications are commonly prescribed, including:

- **Statins:** Statins are broadly used to lesser cholesterol levels in the blood. They prevent the enzyme liable for cholesterol creation in the liver, reducing the risk of atherosclerosis and CardioVascular events. They are frequently prescribed to people with high LDL cholesterol levels or those at high risk of CVD [23].
- **Beta-Blockers:** These drugs reduce the heart's workload and oxygen claim by blocking the action of stress hormones alike adrenaline. Beta-blockers are generally used to cure situations like hypertension, angina (chest pain), and heart failure [26].
- **ACE Inhibitors (Angiotensin-Converting Enzyme Inhibitors):** ACE inhibitors help to lower blood pressure, relax blood vessels, and lessen the workload on the heart. They are regularly given for hypertension and heart failure [27].
- **Antiplatelet Agents:** Medications like aspirin, clopidogrel and Ticagrelor are used to prevent platelet aggregation and lessen the risk of blood clot development, making them valuable in preventing heart spasms and strokes [28, 29].
- **Anticoagulants:** These drugs, such as warfarin and direct oral anticoagulants, are used to inhibit the development of blood clots in situations like atrial fibrillation and venous thromboembolism [30].
- **Diuretics:** Diuretics help to eliminate additional sodium and water from the body, dropping fluid build-up and pulling down blood pressure, which is helpful in heart failure and hypertension [31].

These medications aim to control risk factors, alleviate symptoms, and reduce the likelihood of adverse CardioVascular events.

### 3.2 Lifestyle Modifications (*e.g*., Smoking, Exercise, Diet, Cessation)

Lifestyle modifications are vital to the controlling of CardioVascular disease and play a key part in both avoidance and treatment:

1. **Diet:** A heart-healthy food is categorized by low saturated and decreased salt consumption, trans fats, increased ingestion of fruits and vegetables, and whole grains. This dietary pattern can help manage blood pressure, cholesterol levels, and weight [32].
2. **Exercise:** Regular physical activity contributes to CardioVascular health by improving circulation, reducing blood pressure and aiding in weight management. Aerobic exercises, strength training, and flexibility exercises are all valuable components of an active lifestyle [33].
3. **Smoking Cessation:** Quitting smoking is one of the most effective steps individuals can take to reduce their risk of CVD. Smoking increases the risk of blood clots, damages blood vessels, and influences the increase of atherosclerosis [34].
4. **Alcohol Moderation:** For those who consume alcohol, moderation is advised. Excessive alcohol consumption can lead to heart muscle damage, high blood pressure, and irregular heart rhythms [35].

Lifestyle alterations are often suggested as the first line of defence against CardioVascular disease, and they are particularly important for preventing the disease in high-risk individuals.

### 3.3 Invasive Procedures (*e.g.*, Angioplasty, Bypass Surgery)

In cases where medications and lifestyle modifications are insufficient, or in emergencies, surgical interventions may be necessary:

1. **Angioplasty (Percutaneous Coronary Intervention**): This method comprises the insert of the catheter with a balloon at the tip into a blocked or narrowed coronary artery. Swelling the balloon helps broaden the artery and increase blood flow. Often, a stent is placed to keep the artery open [36, 37].
2. **Coronary Artery Bypass Surgery (CABG**): In Coronary artery bypass surgery, a surgeon makes a new pathway for blood to flow around a blocked or contracted coronary artery. This is typically done by using a segment of a healthy blood vessel from elsewhere in the body [38].

These surgical interventions are often used in those with significant coronary artery disease and can provide immediate relief from chest pain and rise blood movement to the heart muscle.

While these traditional treatment approaches have been effective in managing CardioVascular disease, there is a growing need to explore emerging strategies that can complement or enhance these methods.

## 4 EMERGING THERAPEUTIC STRATEGIES

### 4.1 Genetic and Personalized Medicine

#### 4.1.1 Role of Genetic Markers in Risk Assessment and Treatment

Genetic and personalized medicine are revolutionizing the approach to CardioVascular disease by leveraging an individual's genetic information to assess their risk and tailor treatment. Key points to consider in this context include:

1. **Genetic Risk Assessment:** Advances in genetics have allowed researchers to identify exact genetic markers related to an improved risk of CardioVascular disease. For instance, certain genetic variations may make an individual more susceptible to elevated cholesterol levels, hypertension, or clotting disorders. By analysing a patient's genetic makeup, healthcare providers can better assess their genetic predisposition to CVD [39].
2. **Pharmacogenomics:** Genetic variations also play a role in how individuals metabolize medications. Pharmacogenomics testing can help determine the most effective and safe drug treatments for specific patients, minimizing adverse reactions and optimizing therapeutic outcomes [40].
3. **Early Detection:** Genetic markers can facilitate the early detection of CardioVascular risk, even before clinical symptoms manifest. This early identification can enable timely interventions, such as lifestyle modifications or targeted therapies, to mitigate the risk of heart disease [41].

#### 4.1.2 Personalized Treatment Plans based on Genetic Profiles

Personalized medicine tailor’s treatments to an individual's unique genetic profile, offering the potential for more effective and safer interventions. Key aspects of personalized treatment plans for CardioVascular disease include:

1. **Drug Selection:** Genetic information can guide healthcare providers in selecting the most appropriate medications for a patient based on their genetic response to drugs. This approach can optimize the management of conditions like high cholesterol and high blood pressure [42].
2. **Diet and Lifestyle Recommendations:** Understanding a patient's genetic susceptibility to certain dietary factors, such as salt sensitivity or lipid metabolism, can inform personalized dietary recommendations. Likewise, knowledge of genetic factors related to physical activity and metabolism can help design individualized exercise plans [43, 44].
3. **Risk Stratification:** Genetic profiling can aid in the stratification of patients based on their risk of CardioVascular events. High-risk individuals may benefit from more aggressive preventive measures or closer monitoring, while lower-risk individuals might require less intensive interventions [45].
4. **Patient Engagement:** Personalized medicine empowers patients to take an active role in their healthcare. By understanding their genetic predispositions and the rationale behind their personalized treatment plans, patients are more likely to adhere to their treatment and lifestyle modifications [46].
5. **Ethical and Privacy Considerations:** Personalized medicine raises ethical and privacy concerns related to the handling of genetic data. Privacy protections, informed consent, and secure data management are critical components of implementing personalized medicine in a responsible and ethical manner [47].

The integration of genetic and personalized medicine in CardioVascular disease management holds the promise of more precise risk assessment, early intervention, and individualized treatment plans, ultimately leading to better patient outcomes and a reduction in the burden of CVD. As we continue to explore emerging therapeutic strategies, it is essential to consider the ethical, logistical, and economic aspects of implementing these advancements in clinical practice [48, 49].

### 4.2 Immunotherapies

Immunotherapies are a broad category of medical treatments that harness the body's immune system to resist many diseases, including cancer and autoimmune disorders.

#### 4.2.1 Immune System Modulation for Reducing Inflammation

This aspect of immunotherapy involves using treatments to modulate the immune system in order to reduce inflammation. This is commonly used in the context of autoimmune diseases, where the immune system misguidedly attacks the body's tissues, leading to tissue damage and inflammation. Various medications, such as corticosteroids and disease-modifying antirheumatic drugs (DMARDs), are used to suppress or modify the immune response, thereby preventing further damage and reducing inflammation. These treatments aim to restore a balance in the immune system to alleviate symptoms and improve the quality of life for individuals with autoimmune conditions [50, 51].

#### 4.2.2 Monoclonal Antibodies and Immune Checkpoint Inhibitors

In cancer treatment, this facet of immunotherapy is frequently linked to it. Monoclonal antibodies, crafted in the lab, are proteins meant to pinpoint specific molecules on cancer cells' surfaces. This aids the immune system in recognizing and combating these cancer cells more effectively. Immune checkpoint inhibitors, a form of immunotherapy, hinder specific proteins on cancer or immune cells' surfaces. This action releases the “brakes” on the immune system, enabling it to more forcefully target cancer cells [52, 53].

Some cool meds known as immune checkpoint inhibitors, like nivolumab and pembrolizumab, go after proteins like PD-1 (programmed cell death protein 1) or PD-L1 (programmed cell death ligand 1). These meds have been doing some impressive stuff in different types of cancer, including lung cancer and melanoma. Basically, they help boost the immune system's power to find and kick out those pesky cancer cells [54].

In summary, immunotherapies encompass a wide variety of treatments that either control the immune system to decrease inflammation (as in autoimmune diseases) or activate the immune system to attack diseases such as cancer through the use of monoclonal antibodies and immune checkpoint inhibitors. These therapies represent a major progression in the field of medicine and have shown great potential in increasing patient outcomes in various disease settings [55].

### 4.3 Nanotechnology and Drug Delivery

Nanotechnology plays a crucial role in the field of medicine, particularly in drug delivery and diagnostic applications:

**Nanoparticles for Targeted Drug Delivery:** Nanoparticles are incredibly small particles, often on the nanometer scale, which can be engineered to carry drugs or therapeutic agents. These nanoparticles can be designed to target specific cells or tissues within the body. This targeted drug delivery is advantageous because it reduces the side effects of the treatment while increasing the therapeutic efficacy. For example, in cancer therapy, nanoparticles can be designed to accumulate selectively in tumour tissues, delivering chemotherapy agents directly to the cancer cells while sparing healthy tissues. This reduces the toxic effects of chemotherapy on healthy cells and enhances the treatment's overall effectiveness. Liposomes, micelles, and dendrimers are examples of nanoparticles used in drug delivery [56, 57].

**Nano-Based Imaging and Diagnostic Techniques:** Nanotechnology has also revolutionized medical imaging and diagnostic tools. Nano-based imaging techniques use nanoparticles to enhance contrast in medical imaging. For example, contrast agents containing nanoparticles can be used in computed tomography (CT) or magnetic resonance imaging (MRI) scans to improve the visualization of specific tissues or structures. Additionally, nanoparticles can be engineered to carry diagnostic markers or agents that can be used to detect specific biomarkers associated with diseases. This enables early and precise diagnosis of various medical conditions. Quantum dots, superparamagnetic nanoparticles, and gold nanoparticles are examples of nanoparticles used in imaging and diagnostics [58, 59].

Nanotechnology has opened up new possibilities in medicine, allowing for more precise drug delivery and more sensitive diagnostic procedures. These advances hold great promise for improving the effectiveness of medical treatments and early disease detection, ultimately benefiting patients and healthcare providers [60].

### 4.4 Stem Cell Therapy

Stem cell therapy is a subject of regenerative medicine that uses the unique properties of stem cells to repair and replace damaged or dysfunctional tissues in the body. Here are the two points you've mentioned in the context of stem cell therapy:

Regenerative Medicine Approaches Using Stem Cells: Stem cells have the remarkable ability to progress into various types of specialized cells in the body. In regenerative medicine, stem cells are utilized to replace or repair damaged or degenerated tissues. There are diverse types of stem cells used in these approaches, including persuaded pluripotent stem cells (iPSCs), embryonic stem cells, and adult stem cells. Stem cell therapies can be employed for a wide range of conditions and diseases, containing orthopedic injuries, neurodegenerative disorders, autoimmune diseases, and more. For example, in the case of spinal cord injuries, stem cell transplantation can promote tissue repair and functional recovery [61, 62].

Tissue Engineering for Heart Repair: Heart tissue engineering is a specialized area of regenerative medicine that focuses on repairing and regenerating heart tissue. Stem cells, particularly induced pluripotent stem cells (iPSCs) and cardiac progenitor cells can be used to generate new heart tissue for patients with heart damage, such as after a heart attack. This involves creating functional heart cells in the laboratory and implanting them into the damaged heart tissue to improve its function and restore cardiac health. Tissue engineering for heart repair holds promise for treating heart diseases and conditions that are otherwise challenging to address with traditional treatments [63, 64].

Stem cell therapy and tissue engineering have the potential to revolutionize the treatment of various medical conditions by enabling the regeneration of damaged or degenerated tissues. These approaches offer new avenues for healing and recovery, particularly in cases where conventional therapies may have limited effectiveness [65].

### 4.5 Telemedicine and Remote Monitoring

Telemedicine and remote monitoring technologies have had a profound impact on the management of CardioVascular disease (CVD) by providing new and more efficient ways to monitor patients and deliver healthcare services. Here is an elaboration on the role of telemedicine and remote monitoring in CVD management [66]:

**Continuous Monitoring of Vital Signs:** Telehealth and remote monitoring solutions enable patients to track their vital signs at home, including blood pressure, heart rate, and sometimes even more advanced metrics like oxygen saturation and ECG data. Patients use wearable devices, mobile apps, or other monitoring equipment that automatically transmit data to healthcare providers. This continuous monitoring permits for real-time assessment of the patient's CardioVascular health, offering a more comprehensive and up-to-date view of their condition [67].

**Early Detection of Changes:** By monitoring vital signs continuously, healthcare providers can detect any significant changes or irregularities in a patient's CardioVascular parameters as they occur. For patients with CVD or those at risk, this early detection is crucial. It allows healthcare professionals to identify potential problems, such as a sudden increase in blood pressure or an irregular heartbeat, and intervene promptly before the situation worsens [68].

**Patient Empowerment:** Telemedicine and remote monitoring enable patients to actively participate in their own care. They can better understand their condition, track their progress, and adhere to treatment plans. This increased patient engagement can lead to better self-management of their CVD, including medication adherence, lifestyle modifications, and timely reporting of symptoms [69].

**Reduced Healthcare Costs:** Remote monitoring can reduce the frequency of in-person doctor visits and hospital admissions. This not only lowers healthcare costs but also reduces the burden on healthcare systems. Patients can often manage their condition from the comfort of their own homes, which is particularly valuable for individuals with chronic CVD [70].

**Improved Access to Care:** Telehealth develops access to healthcare services, especially for patients in distant or underserved areas. Patients can consult with cardiologists and other specialists without the need for long-distance travel. This is particularly important for CVD management, as timely access to CardioVascular care can be critical in emergencies [71].

**Customized Care Plans:** Remote monitoring data provides healthcare providers with valuable insights into a patient's condition, allowing them to tailor treatment plans more effectively. For example, if a patient's blood pressure consistently remains elevated, adjustments to their medication regimen or lifestyle recommendations can be made based on the data collected [72].

**Data-Driven Decision-Making:** Telehealth and remote monitoring systems generate a wealth of data. Advanced analytics and machine learning can be employed to analyse this data, recognizing trends and patterns that may not be evident through traditional methods. These insights can inform treatment decisions and allow for personalized care plans [73].

**Reduction in Hospital Readmissions:** CVD patients often face a risk of readmission after discharge. Remote monitoring can help identify patients at higher risk and intervene early to prevent hospital readmissions, which can be costly and disruptive for patients [74].

In summary, telemedicine and remote monitoring have transformed CVD management by providing continuous, real-time monitoring of vital signs, enabling early intervention, reducing healthcare costs, improving access to care, and empowering patients to actively participate in their health. These technologies are valuable tools in the effort to improve outcomes for individuals with CardioVascular disease [75].

### 4.6 Artificial Intelligence and Machine Learning

Machine Learning (ML) and Artificial Intelligence (AI) have become indispensable tools in healthcare, offering a range of applications that can improve patient care, diagnosis, and treatment. Here are the two points mentioned [76]:

**Predictive Analytics and Risk Assessment Using AI:** AI and ML are being used to analyse large volumes of healthcare data to predict patient outcomes and assess risks. These systems can help recognize patients at high risk for various health conditions, as well as CardioVascular diseases, diabetes, and cancer. By analysing electronic health records, diagnostic test results, and patient history, AI models can detect patterns and trends that may not be apparent through traditional methods. This enables healthcare providers to take preventive measures and provide personalized care to high-risk patients. For example, AI can predict the likelihood of a patient developing a specific disease and recommend appropriate interventions, such as lifestyle modifications or early screenings [77, 78].

**Decision Support Systems for Healthcare Professionals:** AI-powered result support systems are intended to assist healthcare professionals in making informed decisions. These systems can provide real-time information, treatment recommendations, and diagnostic assistance. By analyzing patient data and medical literature, AI can offer evidence-based suggestions for diagnosis and treatment. For example, in radiology, AI can analyse medical images (such as MRIs, CT scans, or X-rays) to aid radiologists in identifying abnormalities and making more accurate diagnoses. In oncology, AI can assist oncologists in choosing the most appropriate treatment plan based on a patient's genetic profile and disease characteristics. These decision support systems boost the accuracy and competence of healthcare professionals, ultimately improving patient care [79, 80].

AI and ML are transforming healthcare by enhancing the accuracy of diagnosis and treatment, optimizing resource allocation, and improving patient outcomes. They offer the potential to transform the field of medicine by enabling personalized treatment plans, data-driven decision-making, and better patient care. However, it is important to consider ethical and privacy concerns, as well as the need for rigorous validation and regulation when implementing AI and ML in healthcare.

### 4.7 Lifestyle and Behavioral Interventions

Lifestyle and behavioural interventions are critical for promoting healthier habits and preventing chronic diseases. Here are the two points you have mentioned [81]:

**Innovative Approaches to Encourage Healthier Habits:** Encouraging individuals to adopt and maintain healthier habits is a fundamental aspect of public health and healthcare. Innovative approaches leverage psychology, technology, and creative strategies to motivate behaviour change. Some examples include [82, 83].

**Social Nudging:** Utilizing social and peer pressure to encourage healthier habits. This could involve social media campaigns, competitions, or community challenges to promote healthy behaviours [84].

**Behavioural Economics:** Applying principles of behavioural economics to influence decision-making, such as using incentives, rewards, and choice architecture to guide individuals toward healthier choices [85].

**Digital Health Interventions:** Developing mobile apps, wearables, and online platforms that provide personalized feedback, reminders, and support for adopting and maintaining healthier lifestyles. These interventions can track physical activity, monitor diet, and offer insights into sleep patterns [86].

**- Environmental Changes:** Modifying the physical environment to make healthier choices more accessible, such as designing walkable cities, providing bicycle lanes, or offering healthier food options in schools and workplaces [87].

**Gamification and Behaviour Change Apps:** Gamification includes applying game design elements and principles to non-game contexts, such as health and wellness apps, to make the process of behaviour change more engaging and rewarding. Behaviour change apps often incorporate gamification strategies to motivate users to adopt and maintain healthier habits. Examples include [88]:

**Point Systems:** Users earn points or rewards for completing health-related tasks, such as tracking daily steps, meeting exercise goals, or maintaining a balanced diet.

**Challenges and Competitions:** Creating challenges or competitions where users can compete with friends or other app users to achieve specific health goals.

**Virtual Rewards:** Providing users with virtual badges, trophies, or other symbolic rewards for achieving milestones, such as losing weight, quitting smoking, or hitting fitness targets.

**Progress Tracking:** Allowing users to visually track their progress and see improvements in their health and fitness over time, can be motivating.

Gamification and behavior change apps leverage human psychology and our natural inclination for competition and achievement. They can make the process of adopting and sustaining healthier habits more enjoyable and rewarding, ultimately increasing the likelihood of long-term success [89].

These innovative approaches and gamification strategies are valuable tools in the effort to encourage healthier habits and improve public health outcomes. They play a critical role in addressing lifestyle-related health challenges, such as sedentary behavior, obesity, and unhealthy dietary choices.

### 4.8 Gene Editing and CRISPR Technology

Gene editing, particularly with CRISPR technology, offers significant potential for therapeutic applications and raises important ethical considerations and challenges. Here are the two points mentioned [90]:

#### 4.8.1 Potential Applications for Genetic Correction

Managing Genetic Disorders: Advanced gene-editing tools such as CRISPR-Cas9 can address or alter genetic mutations linked to hereditary illnesses. Examples include cystic fibrosis, sickle cell anemia, and specific types of muscular dystrophy. By accurately editing the flawed genes, we can potentially alleviate the impact of these conditions [91].

**Cancer Therapy:** Gene editing can be employed in cancer treatment to target and modify genes associated with cancer growth and metastasis. For example, researchers are exploring ways to engineer immune cells to improve target and destroy cancer cells [92].

**Biotechnology and Drug Production:** Gene editing can be used to modify microorganisms, such as bacteria and yeast, for the production of biofuels, pharmaceuticals, and industrial chemicals [93].

Basic Research: CRISPR technology is widely employed in scientific research to create genetically modified organisms, allowing scientists to study gene function and disease mechanisms in more detail [94].

**Ethical Considerations and Challenges:**

Off-Target Effects: One major challenge is the potential for off-target effects. Gene editing may inadvertently alter genes that were not intended to be modified, leading to unforeseen consequences. Ensuring precision in editing is a critical concern [95].

Germline Editing: Germline editing, which involves modifying genes in embryos, sperm, or eggs, has raised ethical concerns. Permanent heritable changes could be passed on to future generations, raising questions about the ethics of creating “designer babies.”

**Informed Consent:** Ensuring that individuals understand the risks and benefits of gene editing, especially in clinical applications, and providing informed consent is essential. This is particularly complex when considering germline editing, as the consequences extend beyond the individual [96].

**Equity and Access:** Issues related to the accessibility and affordability of gene editing technologies must be addressed, ensuring that these technologies are not only available to those who can afford them but are distributed equitably.

**Environmental Impact:** The ecological consequences of genetically modified organisms and their potential effects on ecosystems and biodiversity must be thoroughly assessed.

**Dual-Use Concerns:** The possibility of gene editing technology being misused for harmful purposes, such as bioterrorism or illicit enhancement, is a matter of concern [97].

The ethical considerations surrounding gene editing, particularly when it comes to germline editing, require ongoing discussions and the formation of rigorous directing frameworks to ensure the responsible and safe use of this powerful technology. Balancing the potential for genetic correction with ethical concerns and responsible oversight is a critical aspect of advancing the field of gene editing.

### 4.9 Clinical Trials and Case Studies

#### 4.9.1 Summarization of Recent Clinical Trials on Emerging Therapies

1) “The Promoter, a brain-computer interface-assisted intervention to promote upper limb functional motor recovery after stroke: a statistical analysis plan for a the randomized controlled trial”

The promoter study is a well-structured, randomized trial assessing the effectiveness of the promoter scheme, a brain-computer interface (BCI) developed on EEG tools, in improving hand motor recovery post-stroke. The study utilized an advantage design, comparing two equivalent groups receiving motor imagery (MI) training with or without BCI assistance. The primary focus was on the Upper Extremity Fugal-Meyer Assessment (UE-FMA) score, with additional outcomes covering functional, clinical, and user experience aspects. An internal pilot study was planned to evaluate the model size, employing robust statistical methods including group assessments, critical improvement score generation, subgroup analysis, and addressing missing data. The study was focussed on providing strong evidence for the short and long-term success of the promoter method, with a comprehensive statistical analysis plan ensuring clarity in data interpretation and integration into clinical practice [98].

2) “Testing the Effect of a Smartphone App on Hospital Admissions and Sedentary Behavior in Cardiac Rehabilitation Participants: ToDo-CR Randomized Controlled Trial”

In a randomized skillful trial with 120 applicants across three cardiac rehabilitation programs, we investigated the effects of incorporating an inactive movements variation smartphone app (Vireo app and To-do-CR program) beside traditional cardiac restoration on hospital admissions and emergency department (ED) stays over a 12-month period. Whereas the interference group verified potential decreases in costs related to cardiac-related hospital charges and advances in minor outcomes like BMI, waist edge, and excellence of life, the app did not evidence to be an effective or cost-efficient solution in reducing overall hospital admissions or ED visits compared to standard care. The results indicate that focusing solely on addressing sedentary behavior through smartphone apps might not be enough to decrease hospital admissions and enhance outcomes among individuals participating in cardiac rehabilitation [99].

3.) “Early computed tomography coronary angiography and preventative treatment in patients with suspected acute coronary syndrome: A secondary analysis of the RAPID-CTCA trial”

In this research, scientists inspected evidence collected from a broader clinical trial that addressed the claim of early calculated tomography coronary angiography (CTCA) for persons suspected of having serious coronary disease. The objective was to realize the influence of early CTCA on the reference of preventive actions when compared to conventional care.

The study involved 1,743 patients, and individuals who had an initial CTCA were more inclined to receive specific medications like P2Y12 receptor opponent, double antiplatelet, and statin cures, in contrast to those following standard care. Both sets of patients exhibited comparable rises in prescription rates for other preventive treatments. Notably, individuals with obstructive coronary artery disease in the early CTCA group were given extra preventive measures, whereas those with normal coronary arteries saw decreases in certain medications.

These results indicate that performing an early coronary CT angiography (CTCA) enables a more personalized strategy for recommending preventive treatments, taking into account the unique characteristics of coronary artery disease. The customization of treatment during the initial hospitalization for suspected acute coronary syndrome underscores the potential influence of early CTCA in directing precise and tailored medical interventions [100].

4) “Blood Pressure Management After Endovascular Therapy for Acute Ischemic Stroke: The BEST-II Randomized Clinical Trial”

This phase 2 clinical trial, conducted with a randomized, open-label, blinded endpoint design, sought to assess the impracticality of pursuing lower systolic blood pressure (SBP) targets (<140 mm Hg or <160 mm Hg) compared to a higher target (≤180 mm Hg) in patients who had undergone effective endovascular therapy for acute ischemic stroke.

In a group of 120 participants, the research examined the post-treatment size of the infarct and the modified Rankin Scale (mRS) score, adjusted for utility, at 36 hours and 90 days. The outcomes revealed that aiming for lower systolic blood pressure (SBP) targets did not show signs of being ineffective when compared to higher targets. Nevertheless, the results implied a slim chance of deriving benefits from pursuing lower SBP targets in a more extensive future trial, particularly following endovascular therapy [101].

5) “Comparing 5G mobile stroke unit and emergency medical service in patients acute ischemic stroke eligible for t-PA treatment: A prospective, single-center clinical trial in Ya'an, China”

Between February 2020 and January 2022, a research investigation scrutinized the results of treating acute stroke patients using 5G mobile stroke units (MSU) *versus* conventional emergency services. Out of 2,281 patients, 207 eligible for intervention exhibited a superior 90-day recovery rate (82.2%) in the MSU cohort compared to the EMS group (72.6%). The provision of care through MSUs resulted in a more rapid diagnosis and treatment, coupled with decreased hospitalization costs. These findings imply that the utilization of 5G MSU care has the potential to improve outcomes and could potentially supplant traditional stroke treatments, especially with the expansion of digital infrastructure, including 5G technology, in China [102].

6) “RNA interference targeting ANGPTL3 for triglyceride and cholesterol-lowering: phase 1 basket trial cohorts”

In the initial human trial, ARO-ANG3, a treatment designed to target ANGPTL3, demonstrated both safety and beneficial effects on lipoprotein metabolism. The therapy was well-received in both healthy individuals and those with hepatic steatosis, exhibiting fast absorption and a lasting positive influence. Significant reductions in ANGPTL3, triglyceride, and non-HDL cholesterol levels were observed, indicating potential efficacy in treating atherosclerotic cardiovascular disease. The study provides support for ARO-ANG3 as a promising approach for regulating lipid metabolism [103].

7) “Single Ascending Dose Study of a Short Interfering RNA Targeting Lipoprotein(a) Production in Individuals With Elevated Plasma Lipoprotein(a) Levels”

The APOLLO trial investigated SLN360, an siRNA targeting apo(a) production, as a treatment for elevated Lp(a) levels, a known risk factor for cardiovascular disease. In this study of 32 adults with elevated Lp(a) and no known cardiovascular disease, SLN360 showed promising results. It was well-tolerated, with mild adverse events, and led to significant and sustained reductions in Lp(a) levels for up to 150 days post-administration. This suggests that SLN360 could be an effective therapy for individuals with elevated Lp(a), potentially reducing their risk of heart attacks, strokes, and other cardiovascular events. Further research is needed to assess long-term safety, optimal dosing regimens, and impact on cardiovascular outcomes. If successful, SLN360 could provide a valuable treatment option for those at risk due to high Lp(a) levels [104].

#### 4.9.2 Highlight Successful Case Studies and Patient Outcomes

##### 4.9.2.1 “Electronic Visualized Double-lumen Endobronchial Tube for Situs Inversus Totalis: A Case Report and Literature Review”

- **Case Overview**
  - Focuses on anesthesia for a patient with situs inversus totalis (SIT).
  - Utilizes an electronic visualized double-lumen end bronchial tube (E-visual DLT).
- **Challenges and Reversed Anatomy**
  - Faces difficulties due to the reversed anatomy in SIT.
  - Requires a specialized strategy for effective one-lung ventilation during thoracoscopic surgery.
- **Multidisciplinary Approach**
  - Involves a multidisciplinary team in the planning and execution of the anesthesia strategy.
  - Collaboration is essential for addressing anatomical challenges and ensuring patient safety.
- **Anesthesia Technique**
  - Implements left-sided E-visual DLT placement through the right bronchus.
  - Tailored approach to accommodate the unique anatomical features of the patient.
- **Preoperative Preparation**
  - Emphasizes meticulous planning, monitoring, and simulations.
  - Ensures comprehensive preoperative preparation to anticipate and address potential complications.
- **Outcome**
  - Successfully conducts the procedure with no reported complications.
  - Demonstrates the effectiveness of the tailored anesthesia technique for SIT patients [105].

##### ***4.9.2.2 “Treatment of Aneurysmal Artery with PED:*** A Case Report”

- **Patient Profile**
  - Forty-eight-year-old female patient with a continuous frontal headache persisting for three weeks.
- **Diagnosis**
  - Identified with a saccular aneurysm in the right internal carotid artery.
  - Diagnosed through cerebral computed tomographic angiography and magnetic resonance angiography.
- **Confirmation**
  - Digital subtraction angiography further confirms the presence of a sizable saccular aneurysm enveloping the parent artery in the internal carotid artery.
- **Treatment Approach**
  - Treatment involves both a flow diverter stent and coil embolization.
  - Procedure conducted successfully to address the aneurysm.
- **Post-Interventional Care**
  - Patient receives care in the intensive care unit.
  - Dual antiplatelet therapy with aspirin and ticagrelor was administered.
- **Recovery and Discharge**
  - Patient discharged after a 10-day hospital stay.
  - No reported complications following the intervention.
- **Follow-Up and Results**
  - One-year follow-up digital subtraction angiography shows no reappearance of the aneurysm.
  - Positive changes noted in neurological symptoms by the patient.
- **Clinical Significance**
  - Simultaneous use of flow diverter stents and coil embolization in sizable saccular aneurysms is highlighted.
  - Managing aneurysmal parent arteries is discussed, emphasizing clinical importance.
- **Lessons Learned**
  - The case provides valuable insights for managing uncommon events like an aneurysmal parent artery.
  - Serves as a reference for future cases involving comparable situations.
- **Conclusion**
  - Overall, the successful treatment outcome and positive patient response contribute to the clinical understanding of managing complex cases involving saccular aneurysms and aneurysmal parent arteries [106].

##### 4.9.2.3 “Utilizing Pharmacist-Led Telehealth Services in Ambulatory Patients with Heart Failure”

- **Objective**
  - Assess the impact of pharmacist interventions *via* telehealth on heart failure-related hospitalizations in adult patients.
- **Approach**
  - Retrospective and descriptive cohort investigation.
  - Focus on individuals within a single primary care facility.
  - Data collected through electronic chart assessments from January to December 2021.
  - Inclusion criteria: Adults diagnosed with heart failure are recommended for telehealth consultation with a pharmacist.
- **Primary and Secondary Outcomes**
  - Primary focus on the count of hospitalizations related to heart failure post-pharmacist intervention.
  - Secondary emphasis on the tally of hospitalizations linked to cardiovascular issues.
- **Findings**
  - The study involves 37 patients.
  - Following pharmacist intervention, only two patients were hospitalized for heart failure.
  - Fifteen patients were admitted for hospitalizations related to cardiovascular issues.
- **Conclusion**
  - Highlights the use of telehealth services led by pharmacists for individuals with chronic heart failure.
  - Affirms the promising impact of pharmacist-driven telehealth initiatives in reducing hospitalizations associated with heart failure in outpatient settings [107].

##### ***4.9.2.4 “Stem Cell Therapy: A Possible Role in the Treatment of Patients with Chronic Limb-Threatening*** Ischemia?”

- **Study Objective**
  - Aims to present findings from a pilot, multicenter, prospective trial.
  - Focuses on investigating the safety and efficacy of bone marrow aspirate concentrate (BMAC) in individuals with chronic limb-threatening ischemia (CLTI).
- **Patient Profile**
  - 62-year-old male with a medical history including diabetes, hypertension, hyperlipidemia, and ongoing smoking.
  - Presents with a non-healing wound on the right first toe accompanied by dry gangrene.
- **Treatment Decision**
  - Conventional revascularization deemed impractical due to anatomical constraints.
  - Opted for Bone Marrow Aspirate Concentrate (BMAC) treatment.
- **Treatment Outcome**
  - No untoward events were noted during and after BMAC treatment.
  - Subsequent care involves managing osteomyelitis, optimizing medical comorbidities, and wound care.
- **Follow-up Results**
  - At the one-year follow-up, significant improvement in the wound was observed.
  - Avoids the necessity for amputation.
- **Conclusion**
  - The case serves as an illustration of BMAC as an alternative revascularization technique for CLTI patients.
  - Emphasizes the importance of a multidisciplinary approach for optimal outcomes in treating CLTI with BMAC [108].

## 5 CHALLENGES AND BARRIERS

An overview of challenges and barriers in the field of emerging therapies and future prospects in CardioVascular disease management.

### 5.1 Safety and Ethical Concerns with Emerging Therapies

As new therapies, especially in the realm of gene editing and advanced biotechnologies, are developed, there are concerns about unintended consequences, off-target effects, and long-term safety. Ensuring that these therapies are both effective and safe is a major challenge.

Ethical concerns arise in areas like germline editing, where permanent heritable changes are possible. Ensuring that ethical principles, such as informed consent and responsible use, are followed is crucial [109].

### 5.2 Regulatory Hurdles and Approval Processes

Navigating the transition of emerging therapies from the research phase to clinical application is an intricate and meticulously regulated endeavor. Adhering to the rigorous standards set by regulatory bodies like the FDA (U.S. Food and Drug Administration) can prove to be a time-intensive and financially demanding undertaking.

Demonstrating the safety and efficacy of novel therapies through rigorous clinical trials is a major barrier, and navigating the regulatory pathway is challenging for both researchers and pharmaceutical companies.

### 5.3 Accessibility and Cost Issues

Emerging therapies, especially those based on cutting-edge technologies, may be prohibitively expensive and not widely accessible. This creates disparities in healthcare access.

Addressing the cost-effectiveness and affordability of these therapies is essential to ensure that they benefit a broader range of patients [110, 111].

## 6 FUTURE PROSPECTS

### 6.1 Predictions for the Future of Cardiovascular Disease treatment

Personalized Medicine: The future of CVD management will likely involve more personalized treatment approaches, based on genetic, genomic, and lifestyle factors.

Telehealth and Remote Monitoring: The use of telemedicine and remote monitoring technologies will continue to grow, enabling more efficient and patient-centric care for CardioVascular diseases.

AI and Machine Learning: Advanced AI and ML tools will play an increasingly significant role in diagnosing CVD, predicting patient outcomes, and optimizing treatment plans [112-114].

### 6.2 Potential Breakthroughs and Areas for Further Research

● Advanced Therapies: Ongoing research into gene editing, RNA-based therapies, and stem cell treatments may yield breakthroughs in treating and even curing some forms of CardioVascular disease [115].
● Precision Medicine: Continued research into the genetic and molecular underpinnings of CVD will contribute to more targeted and effective therapies [116].
● Health Equity: Addressing disparities in CVD care and outcomes will remain a focus, with research and interventions aimed at reducing inequalities in healthcare access and outcomes [117].
● The future of CardioVascular disease management holds promise for more effective, personalized, and accessible treatments. However, it will require addressing the current challenges and continuing to innovate in research, technology, and healthcare delivery.

## CONCLUSION

Recap of Key Findings and Emerging Therapeutic Strategies: Emerging therapeutic strategies for managing CardioVascular disease (CVD) include gene editing, RNA-based therapies, telehealth and remote monitoring, AI and machine learning, and nanotechnology for targeted drug delivery and imaging. These strategies have the potential to transform CVD management by offering more precise treatment options, refining patient outcomes, and decreasing the burden of the disease. Challenges and ethical considerations in gene editing and advanced therapies need to be addressed through responsible research and regulation. Predictive analytics, personalized medicine, and remote monitoring are shaping the future of CVD management. This review emphasizes the need for a multidisciplinary approach in managing cardiovascular disease: Managing CardioVascular disease requires a collaborative and multidisciplinary method involving geneticists, cardiologists, researchers, dietitians, nurses, and other healthcare professionals. Cardiologists play a central role in diagnosis and treatment, but geneticists can provide awareness of the genetic basis of CVD. Dietitians and lifestyle coaches are essential for promoting healthier habits, which are key to preventing and managing CVD. Researchers and technologists are critical for advancing therapies and tools, such as AI and remote monitoring, that can enhance CVD management. A multidisciplinary method ensures that patients get inclusive care that addresses the medical, genetic, lifestyle, and technological aspects of CVD. In summary, a multidisciplinary approach that leverages emerging therapeutic strategies and emphasizes collaboration among healthcare professionals is essential in effectively managing CardioVascular disease. This approach holds the promise of improving patient outcomes and decreasing the global burden of CVD.

## Figures and Tables

**Table 1:** Descriptions of various cardiovascular diseases.

**Cardiovascular Disease**	**Description**
Coronary Heart Disease	Disease of the blood vessels supplying the heart muscle
Cerebrovascular Disease	Disease of the blood vessels supplying the brain
Peripheral Arterial Disease	Disease of blood vessels supplying the arms and legs
Rheumatic Heart Disease	Damage to the heart muscle and heart valves from rheumatic fever, caused by streptococcal bacteria
Congenital Heart Disease	Birth defects that affect the normal development and functioning of the heart caused by malformations at birth
Deep Vein Thrombosis and Pulmonary Embolism	Blood clots in the leg veins that can dislodge and move to the heart and lungs

**Note:** [5].

## LIST OF ABBREVIATIONS

CVD: Cardiovascular Diseases
ACE: Angiotensin-Converting Enzyme
LDL: Low Density Lipoprotein
DMARDS: Disease Modifying Antirheumatic Drugs
PD-1: Programmed Cell Dead Protein 1
PD-L1: Programmed Cell Dead Ligand 1
CT: Computer Tomography
MRI: Magnetic Resonance Imaging
IPSCs: Induced Pluripotent Stem Cells
ECG: Electrocardiogram
ML: Machine Learning
AI: Artificial Intelligence
CRISPR: Clustered Regularly Interspaced Short Palindromic Repeats
BCI: Brain Computer Interface
EEG: Electroencephalogram
MI: Motor Imagery
UE-FMA: Upper Extremity Fugl-Meyer Assessment
ED: Emergency Department
CTCA: Calculated Tomography Coronary Angiography
SBP: Systolic Blood Pressure
mRS: Modified Ranking Scale
MSU: Mobile Stock Unit
HDL: High Density Lipoprotein
SIT: Situs Inversus Totalis
CLTI: Chronic Limb Threatening Ischemia
BMAC: Bone Marrow Aspirate Concentrate
FDA: Food and Drug Administration
RNA: Ribonucleic Acid
Apo: apolipoprotein
